# The aftereffect of perceived duration is contingent on auditory frequency but not visual orientation

**DOI:** 10.1038/srep10124

**Published:** 2015-06-09

**Authors:** Baolin Li, Xiangyong Yuan, Xiting Huang

**Affiliations:** 1 Key laboratory of cognition and personality (SWU), Ministry of Education, Chongqing 400715, China; 2 Faculty of Psychology, Southwest University, Chongqing 400715, China

## Abstract

Recent sensory history plays a critical role in duration perception. It has been established that after adapting to a particular duration, the test durations within a certain range appear to be distorted. To explore whether the aftereffect of perceived duration can be constrained by sensory modality and stimulus feature within a modality, the current study applied the technique of simultaneous sensory adaptation, by which observers were able to simultaneously adapt to two durations defined by two different stimuli. Using both simple visual and auditory stimuli, we found that the aftereffect of perceived duration is modality specific and contingent on auditory frequency but not visual orientation of the stimulus. These results demonstrate that there are independent timers responsible for the aftereffects of perceived duration in each sensory modality. Furthermore, the timer for the auditory modality may be located at a relatively earlier stage of sensory processing than the timer for the visual modality.

Time is a fundamental aspect of human experience. To ensure our survival, it is necessary for the brain to be sensitive to timing on a variety of scales, from microseconds to circadian cycles. In the current paper, we focus on time perception within the sub-second range, which is essential for many important sensory and perceptual tasks, including speech^1^,^2^, motor coordination^3^,^4^, and multisensory interaction and integration^5^. Although sub-second timing is quite natural in our daily life, its neural bases remains unclear.

Recent sensory history plays a critical role in time perception. Specifically, adaptation to a repeating stimulus of a constant duration induces distortions in perceived duration of subsequently presented test stimuli. For example, after adapting to a long tone (800 or 1000 ms), an intermediate tone (600 ms) appears shorter than it would normally appear; and after adapting to a short tone (200 or 400 ms), the intermediate tone (600 ms) tends to increase its apparent duration^6^. This negative aftereffect of perceived duration has been reproduced in subsequent research^7^,^8^,^9^. Humans live in a cluttered environment where many different stimuli of various durations from different modalities or within a modality can be encountered concurrently. Given these circumstances, a question worth considering is whether the aftereffect of perceived duration is constrained by sensory modality and the stimulus feature within a modality.

Becker and Rasmussen^7^ have found that after adapting to a fast auditory rhythm, a moderately fast test rhythm appeared slower and vice versa for the auditory modality but not for the visual modality, which suggests that the aftereffect of perceived duration is modality specific. Recently, a similar phenomenon was demonstrated by Heron *et al.*^8^, who employed the technique of sensory adaptation and found that the aftereffect of perceived duration appeared to be limited to the adapting modality. However, these results are apparently inconsistent with those reported by Zhang *et al.*^10^. In their experiments, they found that adapting to a short time interval by observing a series of two pairs of blinking discs at the centre of the screen and hearing a sequence of two pairs of beeps led to more reports of ‘group motion’ in the subsequent visual Ternus probe, confirming that the temporal aftereffect generalizes between sensory modalities. So the debate on modality specificity of the aftereffect of perceived duration remains. Further, although the major studies have suggested the aftereffect of perceived duration is modality specific, whether these modality specific adaptation mechanisms can operate simultaneously in parallel with one another is unclear given that there was only one adaptation duration defined by one sensory modality at a time in the adaptation phases of previous studies. Thus, one goal of the present study was to investigate whether people can concurrently obtain two distinct aftereffects of perceived duration for different sensory modalities using the method of category ratings^11^,^12^ and the technique of simultaneous sensory adaptation^13^,^14^,^15^ which simultaneously adapts to opposing visual and auditory durations (Fig. 1). Using the method of category ratings allows for an absolutely unimodal estimate of test duration without requiring any crossmodal comparison, which may have been distorted by the simultaneous adaptation to duration defined by the other modality. Settling the modality specificity of the aftereffect is useful in addressing the neural bases of time perception. If the aftereffect of perceived duration is modality specific and it is possible to concurrently obtain two distinct aftereffects for different sensory modalities, it implies that they are achieved by multiple modality-specific systems. However, if they are controlled by a centralized mechanism, which is independent of the specific sensory modality, then no distinct concurrent aftereffects for different sensory modalities should be expected.

The second goal of the current study was to investigate whether the aftereffect of perceived duration is contingent on the stimulus features within the visual or auditory modality using a duration discrimination task (Fig. 2). According to previous studies^6^,^7^,^8^, the aftereffect of perceived duration can be explained by a model of neural adaptation that has been proposed to account for other aftereffects^16^,^17^,^18^. According to this model, there are duration detectors, each of which responds selectively to a narrow range of stimulus durations centred on its preferred duration, situated in early areas of the visual and auditory nervous systems. Thus, a feasible hypothesis is that these duration detectors could also be sensitive to low-level stimulus features, and the aftereffect of perceived duration could be constrained by these stimulus features. Indeed, this opinion has been supported by the study of Walker and Irion^19^, which has shown that the aftereffect of perceived duration is contingent on pitch, suggesting that some duration detectors are sensitive to the pitch as well as to the duration (but see study of Allan^20^). However, it remains unclear whether the contingent aftereffect of perceived duration occurs in the visual modality, too. Since substantial evidence has indicated that there are modal differences in timing, for example, audition has been found to possess a higher temporal resolution than vision^21^,^22^ and auditory duration usually tends to be judged as longer than visual duration^23^,^24^,^25^, it is necessary to systematically address the contingent aftereffect of perceived duration for both visual and auditory modalities.

Taken together, there are still some unsettled issues concerning the aftereffect of perceived duration. Thus, the present study systematically investigated the influence of modality and stimulus feature on the aftereffect of perceived duration using the technique of simultaneous sensory adaptation, which is useful in realizing the neural bases of time perception.

## Results

### Experiment 1

For each observer, the scores were averaged across the seven test durations to calculate mean score (MS) for each of the four conditions (2 adaptations × 2 test modalities). In order to compare the aftereffects of perceived duration between the two test modalities, we calculated the ‘aftereffect magnitude’ as the arithmetic difference between MS values for each adapting configuration and test modality: aftereffect magnitude = (MS _adapt S_)−(MS _adapt L_). Specifically, for the auditory (visual) modality, the aftereffect magnitude was the arithmetic difference between the MS values, in which subjects rated the durations of the auditory (visual) test stimulus in the ‘VLAS’ (‘VSAL’) and ‘VSAL’ (‘VLAS’) conditions. The aftereffect magnitudes for each participant are shown in Table S1.

First, one-sample 2-tailed t-tests showed that the aftereffect magnitude in audition (mean = 0.94, SEM = 0.1) was significantly larger than zero [*t*(9) = 9.35, *P* < 0.001], and the aftereffect magnitude in vision (mean = 0.39, SEM = 0.098) was also significantly larger than zero [*t*(9) = 3.98, *P* = 0.003] (Fig. 3), verifying that the aftereffect of perceived duration still existed in both visual and auditory modalities even when concurrently adapting to two incongruent durations. Second, a paired-samples 2-tailed t-test showed that the aftereffect magnitude in audition was significantly larger than that in vision [*t*(9) = 3.7, *P* = 0.005]. These findings suggest that the aftereffect of perceived duration represents modality-specific encoding, and that the aftereffect magnitudes in vision and audition are different.

### Experiment 2a

For each observer, the proportion of longer responses to test stimuli for each condition (4 adaptations × 2 test stimuli) was plotted as function of test durations and fitted with a logistic function of the form y = 1/(1 + exp(-(x-x0)/b)) (Fig. 4a,b), where x0 is the test duration value corresponding to the point of subjective equality (PSE; 50% response level on the psychometric function) and b provides an estimate of duration discrimination threshold (approximately half the offset between the 27% and 73% response levels). In this way, PSE values were obtained for all conditions. In order to compare PSE values across conditions, the ‘aftereffect magnitude’ was calculated as the arithmetic difference between PSE values for each adapting configuration and test stimulus: aftereffect magnitude = (PSE _adapt L_)−(PSE _adapt S_). That is, in the congruent adaptation conditions, for the horizontal (vertical) Gabor patch, the aftereffect magnitude was the arithmetic difference between the PSE values of horizontal (vertical) Gabor patch in the ‘HLVL’ and ‘HSVS’ conditions; in the incongruent adaptation conditions, for the horizontal (vertical) Gabor patch, the aftereffect magnitude was the arithmetic difference between the PSE values of horizontal (vertical) Gabor patch in the ‘HLVS’ (‘HSVL’) and ‘HSVL’ (‘HLVS’) conditions. The aftereffect magnitudes for each participant are shown in Table S2.

One-sample 2-tailed t-tests showed that the aftereffect magnitudes of both horizontal Gabor patch [mean = 35.86, SEM = 6.066, *t*(5) = 5.91, *P* = 0.002] and vertical Gabor patch [mean = 34.34, SEM = 6.691, *t*(5) = 5.13, *P* = 0.004] were significantly larger than zero in the congruent adaptation conditions. However, in the incongruent adaptation conditions, there were no significant differences from zero for the aftereffect magnitudes of both horizontal Gabor patch [mean = 2.3, SEM = 1.439, *t*(5) = 1.60, *P* = 0.171] and vertical Gabor patch [mean = 3.65, SEM = 5.997, *t*(5) = 0.61, *P* = 0.569] (Fig. 5a). A 2 × 2 repeated-measures ANOVA (within-subjects design) with two levels of adaptation (congruent, incongruent) and two levels of test stimulus (horizontal Gabor patch, vertical Gabor patch) was run on the aftereffect magnitudes. The main effect of adaptation was significant [*F*(1, 5) = 12.83, *P* = 0.016], showing that the aftereffect magnitude in the congruent adaptation condition was significantly larger than that in the incongruent adaptation condition. However, the main effect of test stimulus [*F*(1, 5) < 0.001, *P* = 0.984] and the interaction [*F*(1, 5) = 0.24, *P* = 0.646] were not significant. These result patterns suggest that the aftereffect of perceived duration is not contingent on the visual orientation.

### Experiment 2b

For each observer, the PSE and aftereffect magnitude were calculated for each condition as in Experiment 2a (Figs. 4c,d and Table S3). One-sample 2-tailed t-tests showed that the aftereffect magnitudes of both 500 Hz pure tone [mean = 54.81, SEM = 1.874, *t*(5) = 29.25, *P* < 0.001] and 2000 Hz pure tone [mean = 52.43, SEM = 7.812, *t*(5) = 6.71, *P* = 0.001] were significantly larger than zero in the congruent adaptation conditions. In addition, in the incongruent adaptation conditions, the aftereffect magnitudes of the 500 Hz pure tone [mean = 50.62, SEM = 10.42, *t*(5) = 4.86, *P* = 0.005] and 2000 Hz pure tone [mean = 45.21, SEM = 5.583, *t*(5) = 8.1, *P* < 0.001] were also significantly larger than zero (Fig. 5b). A 2 × 2 repeated-measures ANOVA confirmed this finding and showed that the main effect of adaptation [*F*(1, 5) = 1.36, *P* = 0.296], the main effect of test stimulus [*F*(1, 5) = 1.09, *P* = 0.345], and the interaction [*F*(1, 5) = 0.09, *P* = 0.774] were not significant. These results suggest that the aftereffect of perceived duration is contingent on the auditory frequency.

## Discussion

In the current study, we provide evidence that people can concurrently obtain two distinct aftereffects of perceived duration for different sensory modalities. This not only confirms the aftereffect of perceived duration is modality specific, but also shows these modality specific adaptation mechanisms can operate simultaneously in parallel with one another. However, this is inconsistent with the study of Zhang *et al.*^10^. One of the greatest differences when comparing these studies is the specific task used to measure time perception. More precisely, an implicit timing task was used in the study of Zhang *et al.*^10^, but explicit timing tasks were used in our own and other studies^7^,^8^. Thus, one possible reason responsible for the different results is that subjects might tend to bind the time information to the sensory modality in the explicit timing tasks but not in the implicit timing tasks. In short, our results support the notion that explicit time information is not coded by a central mechanism, and that there are separate timing mechanisms for the aftereffects of perceived duration within each modality. This assumption is also confirmed by the fact that the aftereffect magnitudes in vision and audition are different.

The other major goal of our study was to investigate the influence of stimulus feature within a modality on the aftereffect of perceived duration. Interestingly, there is a significant difference between the patterns of contingent aftereffect for vision and audition. Specifically, our results revealed that the aftereffect of perceived duration is contingent on auditory frequency but not on visual orientation. How could such a difference arise? Because the designs of Experiment 2a and 2b are identical except for the stimuli used to define the durations, the difference should stem from the perceptual process but not from a decision or motor process.

The patterns of our results are similar to those of a previous study that found that perceptual learning of temporal order could transfer across orientation change but not audio frequency change^26^. Similar to this study, the different contingent aftereffects of perceived duration between vision and audition may be attributed to the site of the duration-tuned neurons. More specifically, the aftereffect of perceived duration is not constrained by orientation, suggesting that duration-tuned neurons for this aftereffect are not sensitive to orientation. Processing in early visual cortex (V1) with its orientation-tuned cells is highly specific for orientation^27^,^28^, which suggests that the duration-tuned neurons in the visual system may receive visual inputs after the stage where these low-level visual features have been extracted. That is, duration-tuned neurons in the visual system may operate after the stage of initial feature coding. Furthermore, for the auditory modality, the result is consistent with the study of Walker and Irion^19^, which demonstrated that the aftereffect of perceived duration is contingent on pitch, suggesting that duration-tuned neurons in the auditory system are sensitive to the frequency of tones. The clear implication of frequency-coded duration detectors is that auditory duration detectors may operate within frequency channels that are likely to be located early in the auditory pathway, possibly even as early as the inferior colliculus which proved to have extremely sharp frequency tuning^29^,^30^. Consistent with this inference are electrophysiological studies that have found duration-tuned neurons in the inferior colliculus^31^,^32^, some of which are also sensitive to echo frequency^33^,^34^. In sum, these patterns of results indicate that duration-tuned neurons in the auditory modality, in contrast to the visual modality, are likely to be situated at a relatively earlier stage of auditory sensory processing.

Regardless of the site of the duration-tuned neurons, there is another explanation for the observed asymmetrical influence of stimulus feature on the aftereffect of perceived duration between vision and audition. From the viewpoint of invariance, changing visual orientation may not be equal to changing audio frequency in the real world. We live in a dynamic environment and our perceptual system is quite flexible. Although visual objects, projected on the retina, frequently change in size, shape, and orientation, we can still recognize them due to perceptual constancy^35^. Furthermore, according to the topological approach to perceptual organization, orientation is a form of Euclidean property whose perceptual salience is low, and its change does not induce the perceptual equivalent of new objects^36^,^37^. Thus, in our experiment, subjects may have perceived the vertical and horizontal Gabor patches as the same object, which can be contrasted with the frequency change. As a basic element of sound, audio frequency is quite stable even under changes in listening position, which is important for us to distinguish auditory stimuli. In the current study, 500 Hz and 2000 Hz pure tones were used, and subjects can easily perceive them as different objects. Therefore, they are likely to bind the different time information to the subjectively different stimuli. Given these distinct modality effects, it is worth considering the issue of stimulus types further. For example, will the visual contingent aftereffect arise if the simple visual stimuli used in our study are replaced by more complex visual stimuli, such as male and female faces? Such an arrangement, which maximizes possible differences between visual stimuli, may facilitate the contingent aftereffect. Thus, whether visual stimulus types will modulate the contingent aftereffect of perceived duration will be decided in future experiments.

Recent studies on temporal adaptation have shown that the temporal-compression aftereffect, induced by adaptation to a flickering (e.g., 20 Hz) visual stimulus and subsequently testing with visual stimulus flickering at a different frequency (e.g., 10 Hz), is related to the magno cells in the lateral geniculate nucleus (LGN)^38^,^39^,^40^,^41^,^42^ (but see studies of Burr and collaborators^43^,^44^). The temporal-dilation aftereffect, induced by a flickering visual adaptor and a static visual test stimulus, is related to cortical visual neurons in V1^45^. The distinct involvement of subcortical (magno cells) and cortical (V1 neurons) visual mechanisms in time perception shows that low-level neurons in the visual system may contribute to time perception, which at first glance might be inconsistent with our finding that the duration-tuned neurons in the visual system may be insensitive to low-level visual features which might be extracted at an early stage. In fact, the aftereffect of perceived duration, which we focused on in our study, is induced by adaptation to the duration itself and shows the ability of recent experience to selectively initiate both expansion and contraction of perceived duration, whereas their aftereffects don’t use any repeated presentation of duration as adaptor and shows unidirectional distortion of perceived duration. In our opinion, the aftereffect in current study is different from theirs and they have no common neural substrate. Apparently, our results, which show that the aftereffect of perceived duration is concurrently limited to the adapted visual and auditory modalities and contingent on the auditory frequency, are not consistent with some other previous findings either. For example, some studies have found that auditory temporal information can asymmetrically affect the processing of visual temporal information^46^,^47^,^48^, and the learning effect in temporal discrimination can transfer to the trained interval presented with tones at untrained audio frequencies^49^,^50^. The possible reason for these inconsistent results is that the multiple clocks in our brain are located at multiple stages of cognitive processing, which can cause different distortion effects in perceiving duration. This consideration has been embodied by the study of Heron *et al.*^9^, which found that there is a neural hierarchy for illusions of time, for example, duration adaptation precedes multisensory integration.

The aim of this study was to systematically examine whether the aftereffect of perceived duration can be constrained by the sensory modality and the stimulus feature within a modality. Exploring these questions is beneficial to elucidate the neural bases of time perception. The results support the assumption that there are independent timers responsible for the aftereffect of perceived duration in each sensory modality. Furthermore, the timer may be at a relatively earlier stage of sensory processing in the auditory modality than that in the visual modality.

## Methods

### Participants

A total of 21 healthy students and the first author took part in the experiments. Ten participants (M = 5, F = 5, age: mean = 21.9, SD = 1.91), who were naive to the purpose of the study, performed in Experiment 1. Another 5 naive participants and the first author (M = 3, F = 3, age: mean = 20.67, SD = 2.25) performed in Experiment 2a, and 6 different naive participants (M = 3, F = 3, age: mean = 21.5, SD = 0.84) performed in Experiment 2b. All participants were right-handed and had normal or corrected-to-normal vision and hearing. They gave informed consent and were paid for their participation. The experiments were conducted in accordance with the Declaration of Helsinki and were approved by the local ethics committee of Southwest University (Chongqing, China).

### Stimuli and apparatus

The visual stimuli consisted of a Gaussian blob (SD = 0.53°, Michelson contrast = 0.74; Experiment 1 and 2b) and Gabor patches (SD = 0.53°, carrier spatial frequency of 1.7c/deg, Michelson contrast = 0.98) oriented horizontally or vertically (Experiment 2a), which were presented on a 22′′ CRT monitor (100 Hz refresh rate, 1024 × 768 pixels; Experiment 1 and 2a) and a 17′′ CRT monitor (85 Hz refresh rate, 1024 × 768 pixels; Experiment 2b) with a grey background (9 cd/m^2^). The viewing distances were set to near 70 cm (Experiment 1 and 2a) and 57 cm (Experiment 2b). The auditory stimuli with a 4-ms fade-in and fade-out consisted of white noise bursts (Experiment 1 and 2a) and 500 Hz and 2000 Hz pure tones (Experiment 2b) at ~60 dB sound pressure level (SPL), which were presented via headphone. Stimuli presentation and data collection were implemented by computer programs designed with E-prime.

### Procedures

**Figure 1:**
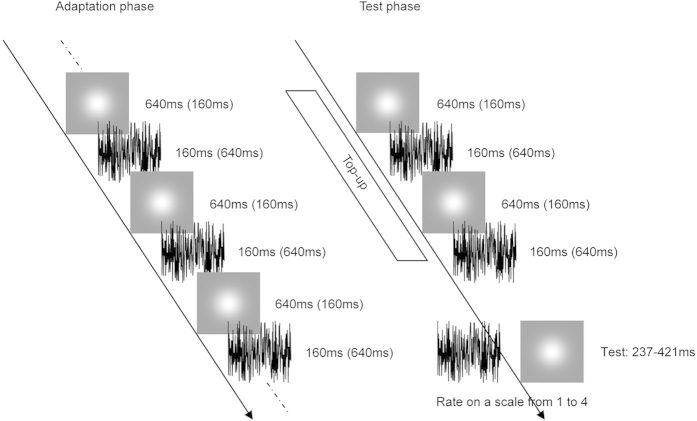
Schematic of the paradigm used in Experiment 1. The visual stimulus was a Gaussian blob and the auditory stimulus was a white noise burst shown in black. Each block started with the adaptation phase consisting of 50 alternating presentations of each of the Gaussian blob and white noise with incongruent durations. Following the adaptation phase, the test phase consisting of four top-up stimuli and a test stimulus (Gaussian blob or white noise) was repeated 84 times. Participants rated the duration of the test stimulus on a scale from 1 (shortest) to 4 (longest) with their right hand once the test stimulus had disappeared.

Experiment 1 included four blocks, each of which consisted of two phases, adaptation and test. During the adaptation phase, subjects observed 50 alternating presentations of the Gaussian blob and white noise with varying durations. Each adapting stimulus was separated by an interval that varied randomly between 500 and 1000 ms. Subjects were instructed to attend to the duration of each adapting stimulus but were not asked to make a perceptual judgment until the test stimulus appeared. Following the adaptation phase, a pause of 2500–3500 ms alerted subjects about the imminent test phase. During the test phase, there was a top-up period consisting of four presentations whose configuration matched that of the adaptation phase. One second later, the test stimulus (Gaussian blob or white noise) was randomly presented, whose duration varied in seven logarithmically spaced steps from 237 to 421 ms (Heron *et al.*^8^). Then, the subjects were asked to rate its duration on a scale from 1 (shortest) to 4 (longest) with their right hand using the computer keyboard after the test stimulus had disappeared (Fig. 1). Once the response occurred, the next top-up-test cycle was triggered automatically after a pause of 1000–2000 ms. There were two adaptation conditions: ‘VLAS’ [visual long (640 ms), auditory short (160 ms)] and ‘VSAL’ [visual short (160 ms), auditory long (640 ms)]. For each adaptation condition, subjects completed two blocks of 84 trials; 42 trials for each of the two test stimuli, with six trials at each of the seven possible test durations. Thus, subjects needed to complete four blocks containing 336 trials, which took about 90 min within a single day. The starting stimulus of the adaptation period (i.e. visual first or auditory first) was counterbalanced across the four blocks. Both the order of trials in a given block and the order of the four blocks were selected by the presentation software in a random manner. To ensure that participants were able to perform the category rating task, they were given two pre-tests before the formal experiment. In the first pre-test with feedback, Gaussian blobs or white noise bursts, whose duration was 200, 300, 400 or 500 ms, were presented and subjects were asked to rate their duration on a scale from 1 (shortest) to 4 (longest) with their right hand once the stimuli disappeared. The second pre-test was similar to the first except that the stimulus durations were as same as those used in the formal experiment and there was no feedback following the response.

**Figure 2:**
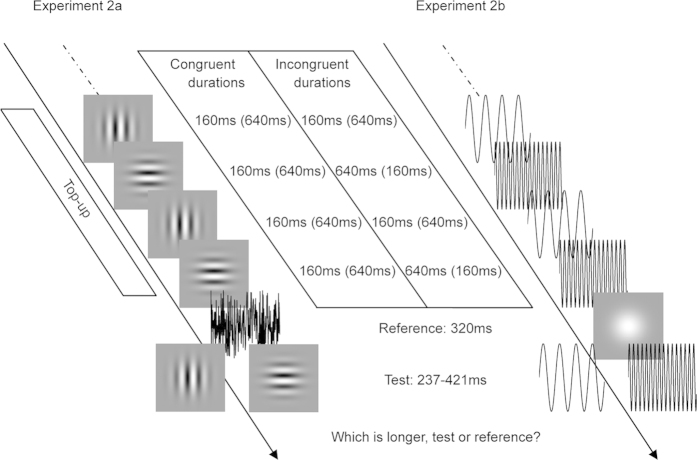
Schematic showing the test phases of Experiment 2a and 2b. In Experiment 2a, both the adaptation and test stimuli were horizontal and vertical Gabor patches, while the reference was a white noise burst shown in black. In Experiment 2b, both the adaptation and test stimuli were high-pitch and low-pitch sounds (2000 Hz and 500 Hz pure tones shown in black), while the reference was a Gaussian blob. Each test trial began with a top-up period in which two repeats of each stimulus configuration, as depicted in the preceding adaptation phase, were repeated. Following the top-up period, the reference lasting 320 ms and test stimulus, whose duration varied in seven logarithmically spaced steps from 237 to 421 ms, were presented successively. Once the test stimulus had disappeared, subjects made an unspeeded, two-alternative forced-choice duration discrimination judgment via the computer keyboard.

**Figure 3:**
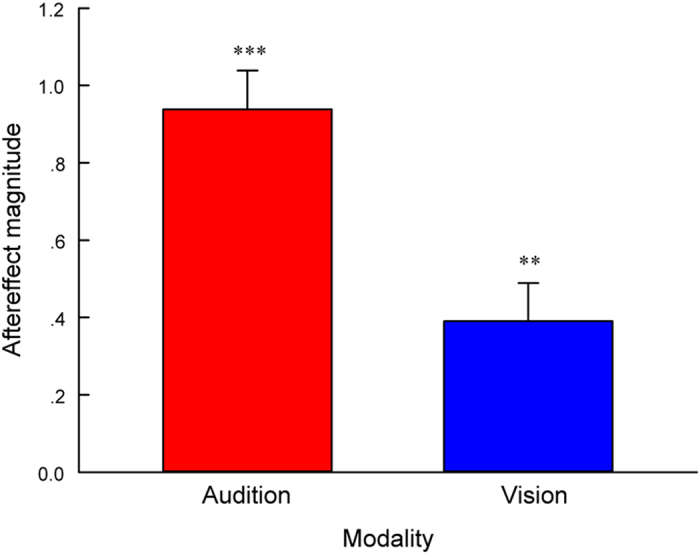
Aftereffect magnitudes averaged across observers (n = 10) for each modality in Experiment 1. Aftereffect magnitude represents the arithmetic difference between mean score values for each adapting configuration and test modality. Error bars represent the SEM across observers. (**P < 0.01; ***P < 0.001)

**Figure 4:**
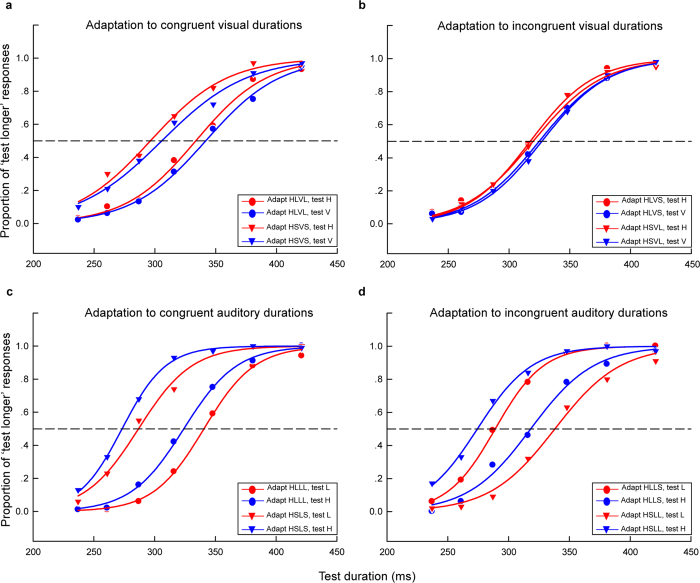
Psychometric functions for six observers showing the proportion of longer responses to test stimuli as a function of test durations in Experiment 2a and 2b. (**a**) Congruent conditions in Experiment 2a where the adaptation durations of horizontal (H) and vertical (V) Gabor patches are the same: ‘both long (HLVL)’ (circles) or ‘both short (HSVS)’ (triangles). (**b**) Incongruent conditions in Experiment 2a where the adaptation durations of horizontal and vertical Gabor patches are different: ‘horizontal long vertical short (HLVS)’ (circles) or ‘horizontal short vertical long (HSVL)’ (triangles). Red and blue curves represent horizontal and vertical test stimuli, respectively, both for the congruent and incongruent conditions in Experiment 2a. (**c**) Congruent conditions in Experiment 2b where the adaptation durations of 500 Hz (L) and 2000 Hz (H) pure tones are the same: ‘both long (HLLL)’ (circles) or ‘both short (HSLS)’ (triangles). (**d**) Incongruent conditions in Experiment 2b where the adaptation durations of 500 Hz and 2000 Hz pure tones are different: ‘2000 Hz long 500 Hz short (HLLS)’ (circles) or ‘2000 Hz short 500 Hz long (HSLL)’ (triangles). Red and blue curves represent 500 Hz and 2000 Hz test stimuli, respectively, both for the congruent and incongruent conditions in Experiment 2b.

**Figure 5:**
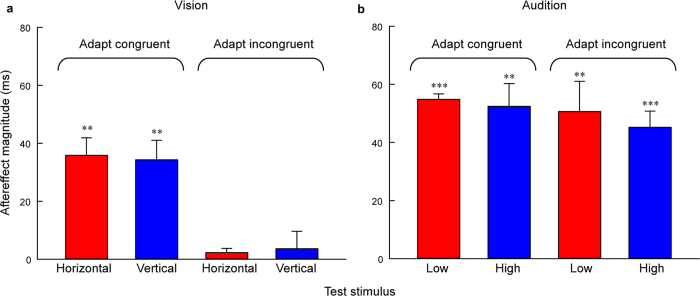
Aftereffect magnitudes averaged across observers for congruent and incongruent adaptation conditions and test stimuli in Experiment 2a and 2b. (**a**) Aftereffect magnitudes in Experiment 2a (vision). Red and blue bars represent horizontal and vertical test stimuli, respectively. (**b**) Aftereffect magnitudes in Experiment 2b (audition). Red and blue bars represent 500 Hz and 2000 Hz test stimuli, respectively. Error bars represent the SEM across observers. (**P < 0.01; ***P < 0.001)

The procedures of Experiment 2a was similar to that of Experiment 1 with the following exceptions. During the adaptation phase, a series of visual stimuli were presented, which comprised 50 alternating presentations of each of the horizontal and vertical Gabor patches with congruent or incongruent durations. After a 2000 ms pause signalling the start of the test phase, four top-up stimuli, which were identical to those presented in the preceding adaptation phase, were presented. Subsequently, a reference and a test stimulus were successively presented. The reference was the white noise that always lasted 320 ms, and the test was the horizontal or vertical Gabor patch whose duration varied in seven logarithmically spaced steps from 237 to 421 ms, which were randomly interleaved using a method of constant stimuli (Heron *et al.*^8^). Subjects were asked to make an unspeeded, two-alternative forced-choice duration discrimination judgment via the computer keyboard (Fig. 2). Half the subjects were told to leave their left hand on the button ‘F’ for ‘test longer than reference’ and their right hand on the button ‘J’ for ‘test shorter than reference’; the other half were told to leave their left hand on the button ‘F’ for ‘test shorter than reference’ and their right hand on the button ‘J’ for ‘test longer than reference’. The inter-stimulus intervals in the test phase and the intervals between two top-up-test cycles varied randomly between 500 and 1000 ms. There were four adaptation conditions: ‘HSVS’ [horizontal and vertical short (160 ms)], ‘HLVL’ [horizontal and vertical long (640 ms)], ‘HSVL’ [horizontal short (160 ms), vertical long (640 ms)] and ‘HLVS’ [horizontal long (640 ms), vertical short (160 ms)]. For each adaptation condition, subjects completed four blocks of 70 test trials with five trials for each of the two visual test stimuli at each of the seven possible durations. Subjects completed four adaptation conditions in a single day, which were repeated over four days, resulting in a total of 1120 trials. The starting stimuli of the adaptation phases (horizontal first or vertical first) were also counterbalanced in which half the subjects observed the sequence ABBA across four days, while the other half observed BAAB (A and B represent horizontal first and vertical first, respectively). The daily experiment began with practice trials until the participant was comfortable in performing the duration discrimination judgment.

The procedures of Experiment 2b were as same as those of Experiment 2a except for the stimuli, that is, the horizontal Gabor patch, vertical Gabor patch, and white noise of Experiment 2a were replaced by the 500 Hz pure tone, 2000 Hz pure tone, and Gaussian blob in Experiment 2b (Fig. 2).

## Additional Information

**How to cite this article**: Li, B. *et al.* The aftereffect of perceived duration is contingent on auditory frequency but not visual orientation. *Sci. Rep.* **5**, 10124; doi: 10.1038/srep10124 (2015).

## Supplementary Material

Supplementary Information
