# Supplementary material for: The aftereffect of perceived duration is contingent on auditory frequency but not visual orientation
Source: Sci Rep. 2015 Jun 9;5:10124. doi: 10.1038/srep10124 (PMC4460570; doi:10.1038/srep10124)
Supplement: Supplementary Information [file srep10124-s1.doc]

The aftereffect of perceived duration is contingent on auditory frequency but not visual orientation

Baolin Li^1,2^, Xiangyong Yuan^1,2^ & Xiting Huang^1,2^,*

1 Key laboratory of cognition and personality (SWU), Ministry of Education, Chongqing 400715, China

2 Faculty of Psychology, Southwest University, Chongqing 400715, China

Corresponding Author: Xiting Huang

Faculty of Psychology

Southwest University

Chongqing 400715, China

Tel: +86 23 6825 3630

Fax: +86 23 6825 3630

E-mail: [**xthuang_swu@163.com**](mailto:xthuang_swu@163.com)

**Table S1.** Aftereffect magnitude for each participant/condition in Experiment 1

| **Experiment 1:**  **Participants** | **Audition** | **Vision** |
| --- | --- | --- |
| 1 | 0.92 | 0.46 |
| 2 | 0.65 | 0.01 |
| 3 | 1.27 | 0.49 |
| 4 | 1.05 | 0.68 |
| 5 | 1.11 | 0.55 |
| 6 | 0.84 | 0.51 |
| 7 | 0.26 | 0.52 |
| 8 | 0.79 | 0.50 |
| 9 | 1.24 | -0.34 |
| 10 | 1.25 | 0.52 |

**Table S2.**  Aftereffect magnitude for each participant/condition in Experiment 2a

| **Experiment 2a: Participants** | **Adaptation to congruent visual durations** | | **Adaptation to incongruent visual durations** | |
| --- | --- | --- | --- | --- |
|  | **Horizontal** | **Vertical** | **Horizontal** | **Vertical** |
| 1 | 44.05 | 40.49 | -0.04 | -5.99 |
| 2 | 19.45 | 23.21 | 2.56 | 10.15 |
| 3 | 14.45 | 18.76 | 5.04 | 29.55 |
| 4 | 43.81 | 37.34 | 7.75 | 4.19 |
| 5 | 44.43 | 23.44 | -0.23 | -7.16 |
| 6 | 48.99 | 62.81 | -1.29 | -8.83 |

**Table S3.** Aftereffect magnitude for each participant/condition in Experiment 2b

| **Experiment 2b: Participants** | **Adaptation to congruent auditory durations** | | **Adaptation to incongruent auditory durations** | |
| --- | --- | --- | --- | --- |
|  | **Low** | **High** | **Low** | **High** |
| 1 | 53.32 | 48.60 | 40.12 | 43.49 |
| 2 | 48.92 | 33.03 | 34.44 | 36.58 |
| 3 | 60.48 | 59.48 | 71.23 | 57.46 |
| 4 | 54.24 | 28.88 | 33.08 | 28.10 |
| 5 | 51.86 | 67.43 | 31.79 | 40.57 |
| 6 | 60.03 | 77.14 | 93.03 | 65.04 |
